# GRATITUDE, RELATIONSHIP QUALITIES, AND LIFE SATISFACTION IN LATE LIFE

**DOI:** 10.1093/geroni/igad104.2190

**Published:** 2023-12-21

**Authors:** Nicole Richards, Zexi Zhou, Karen Fingerman

**Affiliations:** University of Texas at Austin, Austin, Texas, United States; The University of Texas at Austin, Austin, Texas, United States; The University of Texas at Austin, Austin, Texas, United States

## Abstract

Gratitude is associated with better well-being. The Find, Remind, and Bind Theory suggests gratitude may support relationships with greater affection and less conflict, contributing to well-being. This study evaluated gratitude, relationship qualities with social partners, and their association with life satisfaction in late life. We leveraged data from the Daily Experiences and Well-being Study. Older adults aged 65-92 (N = 316, Mean age = 74.03) completed measures of trait gratitude and life satisfaction. They also listed their close social partners and rated their affection and conflict with the 10 closest partners. Linear regression models indicated that older adults who reported higher gratitude also experienced higher levels of life satisfaction. Older adults who rated greater affection in ties to close social partners also experienced higher levels of life satisfaction; conflict was not associated with life satisfaction. We also found moderating effects; less affection with social partners was associated with lower life satisfaction only among older adults with lower gratitude levels. Older adults with higher gratitude levels reported high life satisfaction regardless of affection. Low conflict with social partners was associated with greater life satisfaction for older adults with higher gratitude levels, but participants who experienced high conflict with social partners reported less life satisfaction, regardless of gratitude level. In sum, associations between gratitude and well-being are complex. Gratitude may play a protective role for life satisfaction when older adults navigate their social world without much affection from partners but may be less effective in high conflict contexts.

